# Antibody responses to de novo identified citrullinated fibrinogen peptides in rheumatoid arthritis and visualization of the corresponding B cells

**DOI:** 10.1186/s13075-016-1181-0

**Published:** 2016-12-01

**Authors:** Vijay Joshua, Loes Schobers, Philip J. Titcombe, Lena Israelsson, Johan Rönnelid, Monika Hansson, Anca I. Catrina, Ger J. M. Pruijn, Vivianne Malmström

**Affiliations:** 1 Rheumatology Unit, Department of Medicine, Karolinska Institute, Karolinska University Hospital Solna, 17176 Stockholm, Sweden; 2 Department of Biomolecular Chemistry, Radboud Institute for Molecular Life Sciences and Institute for Molecules and Materials, Radboud University, Nijmegen, Netherlands; 3 Department of Immunology, Genetics and Pathology, Uppsala University, Uppsala, Sweden

**Keywords:** Rheumatoid arthritis, Autoantibodies, Fibrinogen, ACPA, PTPN22

## Abstract

**Background:**

Antibodies against citrullinated proteins (ACPA) are common in patients with rheumatoid arthritis (RA). ACPA can appear before disease onset and target many self-antigens. Citrullinated fibrin/fibrinogen represents a classical ACPA target antigen, and mass spectrometry of RA synovial fluid reveals elevated citrullinated (cit) fibrinogen (Fib) peptides compared to non-RA controls. We investigated the extent to which these less-studied peptides represent autoantibody targets and sought to visualize the corresponding cit-Fib-reactive B cells in RA patients.

**Methods:**

An in-house ELISA was established against four cit-Fib α-subunit peptides (cit-Fib α-35; cit-Fib α-216,218; cit-Fib α-263,271 and cit-Fib α-425,426) and serum from patients with established RA (*n* = 347) and disease controls with psoriatic arthritis (PsA) or ankylosing spondylitis (AS) (*n* = 236) were analyzed. RA patients were genotyped for HLA-DR alleles, PTPN22 R620W and screened for anti-CCP2 and cit-Fib protein antibodies. The cit-Fib peptides were also used to assemble antigen tetramers to identify cit-Fib-reactive B cells in peripheral blood by flow cytometry.

**Results:**

The frequencies of autoantibodies against different cit-Fib epitopes in RA patients compared to PsA/AS patients were: cit-Fib α-35 (RA 20%, vs PsA/AS 1%); cit-Fib α-216,218 (13% vs 0.5%); cit-Fib α-263,271 (21% vs 0.5%) and cit-Fib α-425,426 (17% vs 1%). The presence of autoantibodies against these peptides was associated with presence of anti-CCP2 and anti-cit-Fib protein antibodies. No association was found between HLA-DR shared epitope and antibodies to the different cit-Fib peptides. However, association was observed between the PTPN22 risk allele and positivity to cit-Fib α-35 and cit-Fib α-263,271. B cells carrying surface Ig reactive to these cit-Fib peptides were found in RA peripheral blood and these tend to be more common in PTPN22 risk allele carriers.

**Conclusions:**

Our data show that several cit-Fib peptides are targeted by autoantibodies in RA, but not in PsA/AS, implicating that these are not due to arthritis but more specific for RA etiology. The RA-associated anti-cit protein response is broad with many parallel immune responses. The association between cit-Fib autoantibodies and the PTPN22 R620W risk allele supports the hypothesis of altered B cell regulation, such as autoreactive B cells evading tolerance checkpoints.

## Background

Autoantibodies against citrullinated proteins (ACPA) are specifically associated with rheumatoid arthritis (RA) and are present prior to the onset of the disease [1, 2]. ACPA-positive patients are more likely to suffer from a severe form of the disease with enhanced radiological progression [3]. The core feature of epitopes recognized by ACPA is the non-coding amino acid citrulline (Cit). Accordingly, synthetic (cyclic) citrullinated peptides (CCP) are often used in highly sensitive and specific commercial ELISA kits to detect ACPA [4, 5]. ACPA recognize a variety of citrullinated antigens - prominent among them being citrullinated α-enolase, vimentin, type II collagen, fibrinogen and histone [6, 7].

Fibrinogen, a hexameric molecule containing pairs of α, β and γ chains, is the precursor of fibrin and is involved in the clotting cascade [8]. Citrullinated fibrinogen (cit-Fib) is known to be present in the synovial fluid of patients with RA and autoantibodies against cit-Fib are found both in the sera and synovial fluid of patients with RA [8–10]. Anti-cit-Fib ELISA has been suggested to display similar diagnostic performance to the commercial anti-CCP ELISA [11, 12].

The exact pathophysiological involvement of cit-Fib and anti-cit-Fib antibodies is not fully understood, but there are several indications that they may have a pathogenic role in RA. For instance, it has been demonstrated that immunization of HLA-DR4-IE transgenic mice with cit-Fib results in the induction of (mild) disease symptoms characteristic of RA [13]. Circulating immune complexes containing cit-Fib have been found in patients with RA and these immune complexes have been shown to stimulate macrophages to produce TNF-α via the Fcγ receptor and Toll-like receptor 4 in vitro [14, 15]. Fibrinogen has 81 arginine residues of which two thirds are susceptible to citrullination, although most of these sites do not serve as B cell epitopes [16]. Several studies have identified and validated some of the cit-Fib epitopes in RA using different approaches [8, 16–20]. Cit-Fib has also been verified as an ACPA target in patients with RA throughout the world [21–23] with recognition of the α subunit peptide 36-52 and the β subunit peptide 60-74 being the most prominent.

This study is based on the cit-Fib peptides previously identified via mass spectrometry of RA synovial fluid [24], focusing on the immune reactivity against these less-studied peptides. Using synthetic peptides derived from cit-Fib, we established ELISAs to analyze serum from healthy individuals, patients with RA and patients with non-RA conditions, for the presence of autoantibodies against some of the cit-Fib epitopes. The association between these autoantibodies and the two most prominent genetic RA risk factors, HLA-DR shared epitope (SE) alleles and the PTPN22 R620W allele coding for a tyrosine phosphatase variant, was investigated. Finally, anti-cit-Fib-specific B cells in the peripheral blood of patients with RA were characterized and the association with the aforementioned risk alleles was investigated.

## Methods

### Patients and healthy subjects

Serum samples were collected from 347 patients diagnosed as having established RA according to the ACR criteria [25]. All patients attended the Rheumatology unit at the Karolinska University Hospital, Stockholm, Sweden, where the serum samples were collected and stored at -80 °C until further use. All the samples were previously assayed for anti-CCP2 antibodies and antibodies against full cit-Fib (cit-Fib protein) [10]. Additionally, serum samples from 152 healthy subjects and 236 patients with psoriatic arthritis (PSA) or ankylosing spondylitis (AS) were included as healthy and disease controls, respectively (Table 1). The ethical review board of Karolinska University hospital approved this study and all the patients involved gave informed consent.

**Table 1:** Characteristics of the patients in the different groups

	Healthy	Disease controls	Patients with RA
Number of individuals	152	236	347
Female), *n* (%)	107 (70.4)	115 (48.7)	277 (79.8)
Age, median (range)	57 (23–71)	47 (18–85)	58 (21 − 94)
Anti-CCP2, *n* (%)	5 (3.3)	10 (4.2)	250 (72.0)
Anti-cit-Fib protein, *n* (%)	NA	NA	190 (54.9)

*RA* rheumatoid arthritis, *CCP* cyclic citrullinated peptides, *cit-Fib* citrullinated fibrinogen, *NA* data not available

### HLA-DR and PTPN22 genotyping

A total of 326 of the 347 patients with RA were previously genotyped for the HLA-DR SE allele [6] and 322 of the 347 patients with RA were genotyped for the PTPN22 R620W risk allele [26]. HLA-DRB1*0101, *0102, *0401, *0404, *0405 or *1001 alleles were classified as HLA-shared epitope (HLA-SE) alleles [27].

### ELISA for the detection of IgG against different cit-Fib peptides

Biotinylated Fib peptides (Table 2) were synthesized by a solid-phase procedure using fluorenylmethoxycarbonyl (Fmoc) chemistry as described previously [28]. The peptides were at least 90% pure as deduced from their elution pattern on reversed-phase high performance liquid chromatography (HPLC). Streptavidin-coated high binding capacity 96-well ELISA plates (Thermo Scientific) were coated with the peptides in their native and citrullinated forms at a concentration of 2.5 μg/ml in coating buffer (0.05% Tween-20, 0.1% bovine serum albumin (BSA), Tris-buffered saline). The plates were washed with PBS containing 0.05% Tween-20 after every incubation step. For detection of the antibodies against citrullinated peptides, the serum samples were diluted 1:100 in radioimmunoassay (RIA) buffer (1% BSA, 350 mM NaCl, 10 mM Tris HCl, pH 7.6, 1% (v/v) Triton X-100, 0.5% (weight/volume) sodium deoxycholate, 0.1% sodium dodecyl sulfate). The bound antibodies were detected with horseradish peroxidase-conjugated goat anti-human IgG F(ab’)_2_ (Jackson Immuno Research). Bound antibodies were visualized using the chromogenic substrate 3,3′,5,5′-tetramethylbenzidine (TMB, Sigma-Aldrich). The optical density (OD) was then measured at 450 nm with reference at 650 nm subtracted. A standard curve was included in each plate to convert the OD values into arbitrary units.

**Table 2:** Sequence of the different fibrinogen alpha peptides in their citrullinated form used in the ELISA

Peptide	Amino acid	Peptide sequence
Cit-Fib α 35	29-41	AEGGGV(Cit)GPRVVEZO
Cit-Fib α 216,218	201-225	KDLLPS(Cit)D(Cit)QHLPLIKZO
Cit-Fib α 263,271	256-278	QMRMELE(Cit)PGGNEIT(Cit)GGSTSYGZO
Cit-Fib α 425,426	419-432	NVSPGT(Cit)(Cit)EYHTEKZO

*O* biotin, *Z* 6-aminohexanoic acid

The cutoff value for each of the citrullinated antigens was set to the 98^th^ percentile of values from healthy subjects (*n* = 152). In some cases, samples displayed high reactivity to both a citrullinated peptide and to its native arginine-containing counterpart, thereby distorting the analysis. Therefore, a ratio between the OD values obtained with the peptides containing arginine and citrulline was determined for each sample and samples with a ratio greater than 0.8 were considered negative.

### Tetramer production and flow cytometry

The B cell antigen tetramer consists of an R-phycoerythrin (PE)-labeled streptavidin (SA) core and four identical biotinylated peptides. Tetramers were prepared as previously described [29]. Briefly, the biotinylated cit-Fib peptides used in the ELISA were incubated with SA-PE (Prozyme) at a molar ratio of 10:1. The tetramer fraction was then purified using a 100-kD molecular weight cutoff Amicon Ultra filter (Millipore). The molarity of the tetramer was calculated with the supplier-determined ratio of SA to PE, after measuring the concentration of PE by Nanodrop (Thermo Fischer). The decoy tetramer was prepared, as described above, by incubating the biotinylated native (non-citrullinated) Fib peptides with SA-PE pre-conjugated to Alexa Flour 647 (Molecular Probes Invitrogen). The four α-chain derived Fib peptides were assembled as tetramers separately and then pooled at the time of sample staining. The decoy tetramers were assembled and used in the same manner with the corresponding native Fib peptides.

Tetramer-positive B cells were analyzed in two small cohorts of patients with RA, selected/recruited based on their PTPN22 risk allele status (CC - non risk vs CT - risk). The pilot cohort consisted of cryopreserved peripheral blood mononuclear cells (PBMC) (*n* = 5 individuals, all female, median age 49 (range 40–75), all HLA-DR SE-negative) and the validation cohort consisted of fresh PBMC (*n* = 10 individuals, 7 female/3 male, median age 58 (range 30–69), all HLA-DR SE-positive). PBMC isolated from patients were stained with the decoy and cit-Fib tetramers in buffer containing Fc blocking solution (FcR Blocker Miltenyi Biotec), then passed over a magnetized LS column (Miltenyi Biotec) to enrich for tetramer-binding cells. Both the bound and flow-through fractions were stained with APC-H7-labeled anti-CD3 (SK7), APC-H7-labeled anti-CD14 (MφP9), APC-H7-labeled anti-CD16 (3G8), BV421-labeled anti-CD19 (HIB19), V500-C-labeled anti-CD20 (L27), PE-Cy7-labeled anti-CD27 (M-T271) and FITC-labeled anti-IgD (IA6-2) (all antibodies from BD Biosciences). All the incubation and wash steps were performed using MACS buffer containing 1 mM EDTA and 0.5% BSA in PBS.

Flow cytometry was performed using a 4-laser (405 nm, 488 nm, 561 nm and 640 nm) LSR Fortessa (BD Biosciences) and analyzed with FlowJo software (Tree Star). Fluorescent AccuCheck counting beads (Invitrogen) were used to calculate total numbers of live lymphocytes in the column-bound and flow-through suspensions. The gating strategy for tetramer staining was based on a forward scatter (FSC)/side scatter (SSC) lymphocyte gate and removal of doublets followed by dumping CD3, CD14 and CD16 as depicted in Fig. 3a, before focusing on the B cell subset.

### Statistical analysis

All statistical analyses were carried out using GraphPad Prism (version 6.0) software, SPSS software and Microsoft Excel 2010. Chi square analysis (or Fisher’s exact test when appropriate) was performed to analyze the association between the presence of antibody against the cit-Fib peptides and anti-CCP2 antibodies, antibodies against citrullinated full-length Fib (cit-Fib protein), and HLA-SE and PTPN22 risk alleles. *P* values less than 0.05 were considered significant and have not been corrected for multiple comparisons.

## Results

### Antibodies against the different citrullinated fibrinogen peptides are present in the serum of patients with RA

**Fig. 1:**
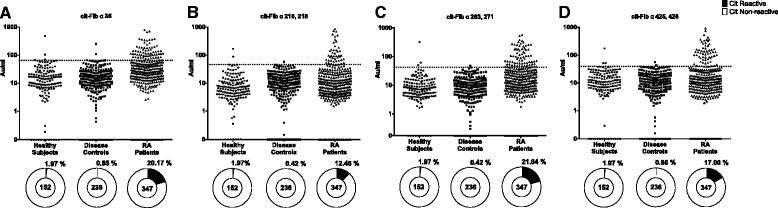
Levels and percentage reactivity of serum antibodies against the different citrullinated fibrinogen (*cit-Fib*) peptides. Comparison of the levels of antibodies against the four different cit-Fib peptides in serum from patients and controls (**a**. cit-Fib α35; **b**. cit-Fib α216;218; **c**. cit-Fib α263, 271; **d**. cit-Fib α425, 426). *Horizontal dotted line* indicates the ELISA cutoff for positivity towards the cit-peptide. *Pie charts* (*bottom*) represent the percentage positivity in each cohort. Number of individuals is indicated in the *centre* of the pie chart. *RA* rheumatoid arthritis

Using mass spectrometry analysis [24], fibrinogen peptides containing the citrulline sites α-35, α-263,271 and α-425,426 had a spectral count 2.5 times higher than controls and were present in the synovial fluid of more patients with RA than controls. All the peptides had a Mascot score greater than 40 and have also been identified by in-vitro citrullination of fibrinogen using human and rabbit PAD enzymes (summarized in [16]). To assess the detailed anti cit-Fib B cell responses, we used ELISA to test for the presence of antibodies against the four different cit-fibrinogen peptides [16, 24]. A cohort of healthy subjects was used to determine the cutoff at the 98^th^ percentile for the ELISA and based upon this cutoff, the cit-Fib reactivity in serum from 347 patients with RA was analyzed. We found relatively weak, though frequently present, reactivity in the RA cohort, with 20.2% towards the cit-Fib α-35, 12.5% towards cit-Fib α-216,218, 21.0% towards cit-Fib α-263,271 and 17.0% towards cit-Fib α-425,426 (Fig. 1).

### Antibodies against the different citrullinated fibrinogen peptides are specific for RA

To understand if the presence of antibodies against these cit-Fib targets were specific for RA, we then analyzed serum from a cohort of patients with non-RA arthritis. For this purpose, we analyzed serum from a cohort of 236 patients with PSA and AS: we only identified a few reactive serum samples, mostly with low levels of the different antibodies. Reactivity against the cit- Fib α-35, cit- Fib α-216,218, cit- Fib α-263,271 and cit- Fib α-425,426 in this cohort was found to be 1.0% (*n* = 2), 0.5% (*n* = 1), 0.5% (*n* = 1) and 1.0% (*n* = 2), respectively (Fig. 1).

### Anti-cit-Fib reactivity is predominantly non-overlapping

**Fig. 2:**
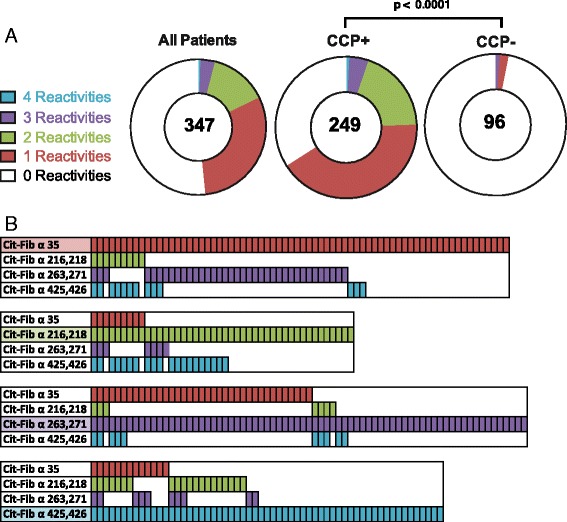
Serum reactivity to multiple citrullinated fibrinogen (*cit-Fib*) epitopes. **a**. *Pie charts* indicate the proportion of serum samples that were reactive with multiple cit-Fib peptides. The total number of individuals is indicated in the *centre* of the pie chart. The *p* value indicates the result from a chi square test comparing the multiple reactivates in anti-citrulline protein antibody (ACPA)-positive vs ACPA-negative individuals. **b**. Illustration of the multiple reactivity seen in individuals positive for antibodies against each cit-Fib peptide. *CCP* cyclic citrullianted peptides

With regard to overlap between the different epitope reactivity, i.e. whether the same patients presented with more than one anti-cit-Fib antibody, and to the distribution compared to anti-CCP2, we observed that most patients were reactive to a single cit-Fib peptide, although some overlap was seen (Fig. 2). Still, the cit-Fib response was predominantly in the anti-CCP2-positive patient subset (*p* < 0.0001).

### Association of anti-cit-Fib with PTPN22 R620W risk allele

It has been previously shown that the presence of ACPA is associated with the HLA-DR SE alleles and with the PTPN22 R620W risk allele [30]. We examined the association between these two genotypes and antibodies against the four cit-Fib peptides, antibodies against whole cit-Fib protein and anti-CCP2 antibodies. The association was significant (*p* ≤ 0.05) for antibodies against the four cit-Fib peptides and anti-CCP2 antibodies (Table 3). Also a significant association was observed between the presence of antibodies against all cit-Fib peptides and those against whole cit-Fib protein. No association was observed between the presence of antibodies against any of the cit-Fib peptides and HLA-DR SE. In contrast, an association was observed between the PTPN22 risk allele and seropositivity for cit-Fib α-35 and cit-fibrinogen α-263,271 (Table 3). PTPN22 risk allele carriers had a significant odds ratio ( (OR) 95% CI) for the presence of antibodies against cit-Fib α-35, OR = 1.8 (1.0–3.1) and cit-Fib α-263,271, OR = 2.0 (1.1–3.4) (Table 4).

**Table 3:** Associations between cit-Fib reactivity and serological (anti CCP2 and anti-cit-Fib) and genetic risk markers (HLA-DR SE and PTPN22 risk allele)

	All patients (*n* = 347)	Anti CCP2 (*n* = 347)			Anti-cit-Fib (*n* = 346)		
		No	Yes	*P* value	No	Yes	*P* value
Cit-Fib α 35	70 (20.2)	0 (0)	70 (28.0)	<0.0001	3 (1.9)	66 (34.74)	<0.0001
Cit-Fib α 216,218	44 (12.7)	2 (2.1)	42 (16.8)	0.0001	13 (8.3)	31 (16.32)	0.03
Cit-Fib α 263,271	73 (21.0)	1 (1.0)	72 (28.8)	<0.0001	8 (5.1)	64 (33.68)	<0.0001
Cit-Fib α 425,426	59 (17.0)	2 (2.1)	57 (22.8)	<0.0001	14 (9)	45 (23.68)	0.0003
Reactivity to at least one cit-Fib peptide	168 (48.4)	3 (3.1)	165 (66.0)	<0.0001	29 (18.6)	138 (72.63)	<0.0001
		HLA-DR SE (*n* = 326)			PTPN22 C1858T (*n* = 322)		
Cit-Fib α 35	70 (20.2)	11 (15.1)	55 (21.74)	0.21	38 (17.4)	28 (27.18)	0.04
Cit-Fib α 216,218	44 (12.7)	12 (16.4)	30 (11.86)	0.30	32 (14.8)	9 (8.74)	0.14
Cit-Fib α 263,271	73 (21.0)	13 (17.8)	57 (22.53)	0.39	39 (17.8)	30 (29.13)	0.02
Cit-Fib α 425,426	59 (17.0)	10 (13.7)	46 (18.18)	0.37	34 (15.5)	23 (22.33)	0.14
Reactivity to at least one cit-Fib peptide	168 (48.4)	34 (46.6)	124 (49.01)	0.71	99 (45.2)	58 (56.31)	0.06
Anti-cit Fib protein	190 (54.9)	29 (40.3)	147 (58.1)	0.01	106 (48.6)	69 (67.0)	0.003

Data represent the numbers (percentage) and *p* values (uncorrected for multiple comparisons). *Cit-Fib* citrullinated fibrinogen, *CCP* cyclic citrullinated peptides, *SE* shared epitope

**Table 4:** Table shows the odds ratio of reactivity against different cit-Fib peptides and genetic risk allels in RA (HLA-DR SE and PTPN22 risk allele)

	Cit-Fib α 35		Cit-Fib α 216,218		Cit-Fib α 263,271		Cit-Fib α 425,426		Cit-Fib protein	
	OR (95% CI)	*P* value	OR (95% CI)	*P* value	OR (95% CI)	*P* value	OR (95% CI)	*P* value	OR (95% CI)	*P* value
SE	1.6 (0.8–3.2)	0.21	0.7 (0.3–1.4)	0.31	1.3 (0.7–2.6)	0.39	1.4 (0.7–2.9)	0.37	2.1 (1.2–3.5)	0.008
PTPN22	1.8 (1–3.1)	0.04	0.6 (0.3–1.2)	0.14	2.0 (1.1–3.4)	0.02	1.6 (0.9–2.8)	0.14	2.1 (1.3–3.5)	0.002

*cit-Fib* citrullinated fibrinogen, *RA* rheumatoid arthritis, *SE* shared epitope

### Visualization of cit-Fib-reactive B cells by tetramer technology

**Fig. 3:**
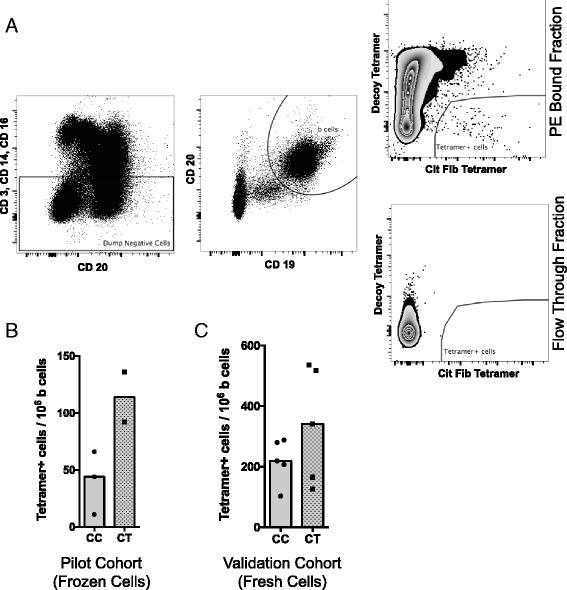
B cell antigen tetramer analysis in individuals with PTPN22 risk allele. **a**. Gating strategy for identification of citrullinated fibrinogen (*cit-Fib*) (pooled tetramers from four α-chain-derived Fib peptides) reactive B cells from peripheral blood mononuclear cells (PBMC). Cells were gated on forward scatter /side scatter to select for lymphocytes and removal of doublets followed by dumping of CD3^+^ CD14^+^ and CD16^+^ cells. All remaining tetramer analysis was performed on CD19^+^ CD20^+^ B cell subsets. **b**. Cit-Fib tetramer analysis in PBMC (frozen) from 5 patients with rheumatoid arthritis (RA), who served as a pilot cohort. *CC* PTPN22 risk allele non-carriers, *CT* risk carriers. Median difference between the two groups was not statistically significant. **c**. Cit-Fib tetramer analysis in PBMC (fresh) from 10 patients with RA, who served as a validation cohort. Median difference between the two groups was not statistically significant

Based on data suggesting a role for PTPN22 in the negative selection of autoreactive B cells [31], we hypothesized that PTPN22 risk allele carriers in our cohort may have an expanded population of cit-Fib reactive B cells relative to non-risk allele carriers. To test this, we constructed B cell antigen tetramers for quantification and comparison of tetramer-positive B cells in PTPN22 risk allele non-carrier (CC) and carrier (CT, TT) patients with RA. Only CD19^+^ CD20^+^ B cells were included in the tetramer analyses (Fig. 3a). Analysis of frozen PBMC from five patients with RA (three CC and two CT) showed a trend in increased frequency of tetramer-positive B cells in the patients carrying the PTPN22 risk allele (Fig. 3b). To validate this finding, we recruited ten additional patients with RA (five CC and five CT PTPN22 allele carriers) and found a similar trend for tetramer-positive B cells with individuals carrying the PTPN22 risk allele (Fig. 3c).

## Discussion

Citrullination of proteins is a posttranslational modification that in susceptible individuals may lead to immune activation, autoantibody production and eventually development of RA [22]. Detection of these antibodies is generally performed with commercial kits developed as diagnostic tools to catch a large array of ACPA specificities. Growing evidence however indicates that a more refined characterization of the ACPA response is needed to define disease subgroups with distinct clinical phenotypes and genetic associations [32]. Here, we characterized serum reactivity against four cit-Fib peptides originally identified by mass-spectrometry analysis of RA synovial fluid. The patient samples represented long-standing RA in need of joint effusions, and as a clinical comparison we used serum samples from equally long-standing psoriatic or spondyloarthritis at time points when patients underwent joint effusions. Antibodies against these citrullinated peptides were specific for RA and show distinct genetic association with PTPN22 but not HLA-SE risk alleles. The data generated represent established RA, and earlier disease stages were not analyzed in the present study.

Citrullinated fibrinogen is one of the first and most well-characterized autoantigens in RA [33]. During the last few years, several new citrullinated proteins have been identified and added to the growing list of potential autoantigens in RA [34–38]. Fibrinogen is elevated in the serum of patients with RA compared to controls [39] and elevated cit-Fib that is citrullinated on fibrin(ogen) has been identified using different techniques, and some of them have been shown to contain ACPA-targeted epitopes [8, 17–19, 40]. Using unbiased mass spectrometry, we have previously identified several cit-Fib peptides as potential antigenic epitopes in RA [24]. We were able to identify serum autoantibodies directed against these citrullinated peptides in patients with RA but not in other types of chronic inflammatory joint disease.

Identification of multiple distinct B cell epitopes recognized by the immune system suggests that loss of B cell tolerance towards cit-Fib might play an important role in development of the disease. The antibody reactivity in the present study was relatively less frequent, but was confined to the seropositive (CCP) RA and provides an addition to the growing family of ACPAs. Their low frequency excludes the possibility of substantiating any clinical and pathological importance at this stage, but instead sheds light on the dysregulated B cell compartment in seropositive RA. A limitation of our study is related to exclusion of those samples reacting with the native peptide that might result in underestimation of the real frequency of these antibodies in patients with RA. This dual reactivity to native and citrullinated self-antigen could indeed be biologically significant and relevant to RA pathogenesis. Further analysis is needed in order to clarify this issue.

Previous reports have shown that antibodies against cit-vimentin and cit-α-enolase and to a lesser extent cit-Fib are associated with presence of HLA-DR shared epitope [22]. We were not able to identify any association between autoantibody reactivity towards our fibrinogen peptides and the HLA-DR shared epitope. Taken together, these data may suggest that ACPA towards some cit-Fib antigens might not arise via classical T cell help to autoreactive B cells, but instead in a more innate or T cell independent fashion. In this context it has been demonstrated that the autoimmune risk allele PTPN22 promotes survival of autoreactive B cells in both RA and type-1 diabetes mellitus by evading the tolerance checkpoints [31, 41, 42].

More recently, PTPN22 and TNF receptor-associated factor (TRAF)3 have been put forward as inhibitors of IL-6R signaling in B cells and plasma cell differentiation [43]. It is intriguing to think that the functional polymorphism in PTPN22 could influence this pathway. Obviously, the association between PTPN22 and the autoantibodies we study could also be indirect; the PTPN22 risk allele has been extensively studied in T cell function and could influence the number of follicular helper T cells, which could subsequently increase B cells and antibody production, as demonstrated in mice [44]. We observed association between the PTPN22 risk allele and autoantibodies against two of the cit-Fib epitopes, although our analysis may have been underpowered to find association for the remaining two sub-specificities. Additional factors directly stimulating B cells to produce antibodies, such as B cell activating factor (BAFF, a.k.a. BLyS) [45] or type I IFN [46] might also play a role, but were not analyzed here.

Historically, ELIspot has been the assay of choice to enumerate and visualize antigen-specific B cells, and represents a very sensitive assay system. More recently, the wish to isolate the antigen-specific B cells has led to development of complementary technologies based on flow cytometry. One setup is based on antigens being coupled to beads and then used to stain B cells [47], and in another setup large antigens could be directly coupled to fluorochromes and used as staining reagents [48, 49]; last, for peptide-antigens, tetramerization of biotinylated peptides before labeling to create so-called B cell tetramers has been utilized [50]. The parallel use of a decoy tetramer allows the purging of false-positive events making this technology robust, and this approach has already been employed to study cit-specific B cells in RA [51, 52].

Utilizing B cell tetramer technology in the present study, we were able to identify cit-Fib reactive B cells in patients with RA and with a trend to higher frequency in PTPN22 risk allele carriers. Though the data were not statistically significant and were limited by there being few data points (frozen cohort, *n* = 5 and fresh cohort, *n* = 10), nevertheless they show a trend that was reproducible in two independent analyses (frozen and fresh cohort). This is in line with previous observations showing the presence of more autoreactive B cells in PTPN22 risk allele carriers with RA and type-1 diabetes mellitus [31]. In this study we used pooled tetramers from the four cit-Fib antigens, because the limited availability of cells from each patient did not allow us to use them separately. Therefore, our data do not provide information on the relative number of the different cit-Fib reactive B cells in each patient sample. Clearly, the technology can also be utilized to study more common ACPA specificities in RA, but that was outside the scope of this study.

## Conclusion

In this study we have extended the family of ACPA targeting citrullinated fibrinogen. Four minor B cell epitopes on the alpha chain of fibrinogen were validated, and patients with RA displayed mainly non-overlapping immune reactivity to these. The RA genetic risk factor PTPN22 was associated with the new cit-Fib reactivity. We also demonstrated the feasibility of visualizing antigen-specific B cells by tetramer technology, which opens up the path for isolation and further characterization of the autoimmune B cells response in RA.

## Abbreviations

ACPA: anti-citrulline protein antibodies
AS: ankylosing spondylitis
BSA: bovine serum albumin
CCP2: cyclic citrullinated peptides 2
Cit: citrulline
cit-Fib: citrullinated fibrinogen
ELISA: enzyme-linked immunosorbent assay
Fib: fibrinogen
HLA: human leucocyte antigen
HPLC: high-performance liquid chromatography
IFN: interferon
IL: interleukin
OD: optical density
PBMC: peripheral blood mononuclear cells
PBS: phosphate-buffered saline
PE: R-phycoerythrin
PsA: psoriatic arthritis
PTPN22: protein tyrosine phosphatase, non-receptor type 22
RA: rheumatoid arthritis
RIA: radioimmunoassay
SA: streptavidin
SE: shared epitope
TMB: 3,3′,5,5′-tetramethylbenzidine
TNF: tumor necrosis factor

## References

[1] Rantapää-Dahlqvist S, de Jong BA, Berglin E, Hallmans G, Wadell G, Stenlund H, Sundin U, van Venrooij WJ (2003). Antibodies against cyclic citrullinated peptide and IgA rheumatoid factor predict the development of rheumatoid arthritis. Arthritis Rheum.

[2] Nielen MM, van Schaardenburg D, Reesink HW, van de Stadt RJ, van der Horst-Bruinsma IE, de Koning MH, Habibuw MR, Vandenbroucke JP, Dijkmans BA (2004). Specific autoantibodies precede the symptoms of rheumatoid arthritis: a study of serial measurements in blood donors. Arthritis Rheum.

[3] Klareskog L, Catrina AI, Paget S (2009). Rheumatoid arthritis. Lancet.

[4] Schellekens GA, de Jong BA, van den Hoogen FH, van de Putte LB, van Venrooij WJ (1998). Citrulline is an essential constituent of antigenic determinants recognized by rheumatoid arthritis-specific autoantibodies. J Clin Invest.

[5] Schellekens GA, Visser H, de Jong BA, van den Hoogen FH, Hazes JM, Breedveld FC, van Venrooij WJ (2000). The diagnostic properties of rheumatoid arthritis antibodies recognizing a cyclic citrullinated peptide. Arthritis Rheum.

[6] Snir O, Widhe M, von Spee C, Lindberg J, Padyukov L, Lundberg K, Engström A, Venables PJ, Lundeberg J, Holmdahl R (2009). Multiple antibody reactivities to citrullinated antigens in sera from patients with rheumatoid arthritis: association with HLA-DRB1 alleles. Ann Rheum Dis.

[7] Pratesi F, Dioni I, Tommasi C, Alcaro MC, Paolini I, Barbetti F, Boscaro F, Panza F, Puxeddu I, Rovero P, et al. Antibodies from patients with rheumatoid arthritis target citrullinated histone 4 contained in neutrophils extracellular traps. Ann Rheum Dis. 2014;73:1414–22. 10.1136/annrheumdis-2012-202765 23727635

[8] Sebbag M, Moinard N, Auger I, Clavel C, Arnaud J, Nogueira L, Roudier J, Serre G (2006). Epitopes of human fibrin recognized by the rheumatoid arthritis-specific autoantibodies to citrullinated proteins. Eur J Immunol.

[9] Takizawa Y, Suzuki A, Sawada T, Ohsaka M, Inoue T, Yamada R, Yamamoto K (2006). Citrullinated fibrinogen detected as a soluble citrullinated autoantigen in rheumatoid arthritis synovial fluids. Ann Rheum Dis.

[10] Snir O, Widhe M, Hermansson M, von Spee C, Lindberg J, Hensen S, Lundberg K, Engström A, Venables PJ, Toes RE (2010). Antibodies to several citrullinated antigens are enriched in the joints of rheumatoid arthritis patients. Arthritis Rheum.

[11] Nielen MM, van der Horst AR, van Schaardenburg D, van der Horst-Bruinsma IE, van de Stadt RJ, Aarden L, Dijkmans BA, Hamann D (2005). Antibodies to citrullinated human fibrinogen (ACF) have diagnostic and prognostic value in early arthritis. Ann Rheum Dis.

[12] Vander Cruyssen B, Cantaert T, Nogueira L, Clavel C, De Rycke L, Dendoven A, Sebag M, Deforce D, Vincent C, Elewaut D (2006). Diagnostic value of anti-human citrullinated fibrinogen ELISA and comparison with four other anti-citrullinated protein assays. Arthr Res Ther.

[13] Hill JA, Bell DA, Brintnell W, Yue D, Wehrli B, Jevnikar AM, Lee DM, Hueber W, Robinson WH, Cairns E (2008). Arthritis induced by posttranslationally modified (citrullinated) fibrinogen in DR4-IE transgenic mice. J Exp Med.

[14] Zhao X, Okeke NL, Sharpe O, Batliwalla FM, Lee AT, Ho PP, Tomooka BH, Gregersen PK, Robinson WH (2008). Circulating immune complexes contain citrullinated fibrinogen in rheumatoid arthritis. Arthritis Res Ther.

[15] Sokolove J, Zhao X, Chandra PE, Robinson WH (2011). Immune complexes containing citrullinated fibrinogen costimulate macrophages via Toll-like receptor 4 and Fcgamma receptor. Arthritis Rheum.

[16] van Beers JJ, Raijmakers R, Alexander LE, Stammen-Vogelzangs J, Lokate AM, Heck AJ, Schasfoort RB, Pruijn GJ (2010). Mapping of citrullinated fibrinogen B-cell epitopes in rheumatoid arthritis by imaging surface plasmon resonance. Arthritis Res Ther.

[17] Matsuo K, Xiang Y, Nakamura H, Masuko K, Yudoh K, Noyori K, Nishioka K, Saito T, Kato T (2006). Identification of novel citrullinated autoantigens of synovium in rheumatoid arthritis using a proteomic approach. Arthritis Res Ther.

[18] Perez ML, Gomara MJ, Ercilla G, Sanmarti R, Haro I (2007). Antibodies to citrullinated human fibrinogen synthetic peptides in diagnosing rheumatoid arthritis. J Med Chem.

[19] Hermansson M, Artemenko K, Ossipova E, Eriksson H, Lengqvist J, Makrygiannakis D, Catrina AI, Nicholas AP, Klareskog L, Savitski M (2010). MS analysis of rheumatoid arthritic synovial tissue identifies specific citrullination sites on fibrinogen. Proteomics Clin Appl.

[20] Hansson M, Mathsson L, Schlederer T, Israelsson L, Matsson P, Nogueira L, Jakobsson PJ, Lundberg K, Malmström V, Serre G (2012). Validation of a multiplex chip-based assay for the detection of autoantibodies against citrullinated peptides. Arthritis Res Ther.

[21] Cornillet M, Sebbag M, Verrouil E, Magyar A, Babos F, Ruyssen-Witrand A, Hudecz F, Cantagrel A, Serre G, Nogueira L (2014). The fibrin-derived citrullinated peptide beta60-74Cit(6)(0), (7)(2), (7)(4) bears the major ACPA epitope recognised by the rheumatoid arthritis-specific anticitrullinated fibrinogen autoantibodies and anti-CCP2 antibodies. Ann Rheum Dis.

[22] Lundberg K, Bengtsson C, Kharlamova N, Reed E, Jiang X, Kallberg H, Pollak-Dorocic I, Israelsson L, Kessel C, Padyukov L (2013). Genetic and environmental determinants for disease risk in subsets of rheumatoid arthritis defined by the anticitrullinated protein/peptide antibody fine specificity profile. Ann Rheum Dis.

[23] Nogueira L, Cornillet M, Singwe-Ngandeu M, Viatte S, Bas S, Gabay C, Serre G (2014). In Black Africans with rheumatoid arthritis, ACPA recognize citrullinated fibrinogen and the derived peptides alpha36-50Cit38,42 and beta60-74Cit60,72,74, like in Caucasians. Clin Immunol.

[24] Raijmakers R, van Beers JJ, El-Azzouny M, Visser NF, Bozic B, Pruijn GJ, Heck AJ (2012). Elevated levels of fibrinogen-derived endogenous citrullinated peptides in synovial fluid of rheumatoid arthritis patients. Arthritis Res Ther.

[25] Arnett FC, Edworthy SM, Bloch DA, McShane DJ, Fries JF, Cooper NS, Healey LA, Kaplan SR, Liang MH, Luthra HS (1988). The American Rheumatism Association 1987 revised criteria for the classification of rheumatoid arthritis. Arthritis Rheum.

[26] Snir O, Gomez-Cabrero D, Montes A, Perez-Pampin E, Gomez-Reino JJ, Seddighzadeh M, Klich KU, Israelsson L, Ding B, Catrina AI (2014). Non-HLA genes PTPN22, CDK6 and PADI4 are associated with specific autoantibodies in HLA-defined subgroups of rheumatoid arthritis. Arthritis Res Ther.

[27] Raychaudhuri S, Sandor C, Stahl EA, Freudenberg J, Lee HS, Jia X, Alfredsson L, Padyukov L, Klareskog L, Worthington J (2012). Five amino acids in three HLA proteins explain most of the association between MHC and seropositive rheumatoid arthritis. Nat Genet.

[28] Hiemstra HS, Duinkerken G, Benckhuijsen WE, Amons R, de Vries RR, Roep BO, Drijfhout JW (1997). The identification of CD4+ T cell epitopes with dedicated synthetic peptide libraries. Proc Natl Acad Sci U S A.

[29] Taylor JJ, Martinez RJ, Titcombe PJ, Barsness LO, Thomas SR, Zhang N, Katzman SD, Jenkins MK, Mueller DL (2012). Deletion and anergy of polyclonal B cells specific for ubiquitous membrane-bound self-antigen. J Exp Med.

[30] Kallberg H, Padyukov L, Plenge RM, Rönnelid J, Gregersen PK, van der Helm-van Mil AH, Toes RE, Huizinga TW, Klareskog L, Alfredsson L (2007). Gene-gene and gene-environment interactions involving HLA-DRB1, PTPN22, and smoking in two subsets of rheumatoid arthritis. Am J Hum Genet.

[31] Menard L, Saadoun D, Isnardi I, Ng YS, Meyers G, Massad C, Price C, Abraham C, Motaghedi R, Buckner JH (2011). The PTPN22 allele encoding an R620W variant interferes with the removal of developing autoreactive B cells in humans. J Clin Invest.

[32] Klareskog L, Lundberg K, Malmström V (2013). Autoimmunity in rheumatoid arthritis: citrulline immunity and beyond. Adv Immunol.

[33] Masson-Bessiere C, Sebbag M, Girbal-Neuhauser E, Nogueira L, Vincent C, Senshu T, Serre G (2001). The major synovial targets of the rheumatoid arthritis-specific antifilaggrin autoantibodies are deiminated forms of the alpha- and beta-chains of fibrin. J Immunol.

[34] Vossenaar ER, Despres N, Lapointe E, van der Heijden A, Lora M, Senshu T, van Venrooij WJ, Menard HA (2004). Rheumatoid arthritis specific anti-Sa antibodies target citrullinated vimentin. Arthritis Res Ther.

[35] Kinloch A, Tatzer V, Wait R, Peston D, Lundberg K, Donatien P, Moyes D, Taylor PC, Venables PJ (2005). Identification of citrullinated alpha-enolase as a candidate autoantigen in rheumatoid arthritis. Arthritis Res Ther.

[36] Burkhardt H, Sehnert B, Bockermann R, Engström A, Kalden JR, Holmdahl R (2005). Humoral immune response to citrullinated collagen type II determinants in early rheumatoid arthritis. Eur J Immunol.

[37] van Beers JJ, Schwarte CM, Stammen-Vogelzangs J, Oosterink E, Bozic B, Pruijn GJ (2013). The rheumatoid arthritis synovial fluid citrullinome reveals novel citrullinated epitopes in apolipoprotein E, myeloid nuclear differentiation antigen, and beta-actin. Arthritis Rheum.

[38] Schwenzer A, Jiang X, Mikuls TR, Payne JB, Sayles HR, Quirke AM, Kessler BM, Fischer R, Venables PJ, Lundberg K, et al. Identification of an immunodominant peptide from citrullinated tenascin-C as a major target for autoantibodies in rheumatoid arthritis. Ann Rheum Dis. 2015;75(10):1876–83. 10.1136/annrheumdis-2015-208495 PMC5036245 26659718

[39] Rooney T, Scherzer R, Shigenaga JK, Graf J, Imboden JB, Grunfeld C (2011). Levels of plasma fibrinogen are elevated in well-controlled rheumatoid arthritis. Rheumatology (Oxford).

[40] Kubota K, Yoneyama-Takazawa T, Ichikawa K (2005). Determination of sites citrullinated by peptidylarginine deiminase using 18O stable isotope labeling and mass spectrometry. Rapid Commun Mass Spectrom.

[41] Giltiay NV, Chappell CP, Clark EA (2012). B-cell selection and the development of autoantibodies. Arthritis Res Ther.

[42] Gianchecchi E, Crino A, Giorda E, Luciano R, Perri V, Russo AL, Cappa M, Rosado MM, Fierabracci A (2014). Altered B cell homeostasis and toll-like receptor 9-driven response in type 1 diabetes carriers of the C1858T PTPN22 allelic variant: implications in the disease pathogenesis. PLoS One.

[43] Lin WW, Yi Z, Stunz LL, Maine CJ, Sherman LA, Bishop GA (2015). The adaptor protein TRAF3 inhibits interleukin-6 receptor signaling in B cells to limit plasma cell development. Sci Signal.

[44] Maine CJ, Marquardt K, Cheung J, Sherman LA (2014). PTPN22 controls the germinal center by influencing the numbers and activity of T follicular helper cells. J Immunol.

[45] Hase H, Kanno Y, Kojima M, Hasegawa K, Sakurai D, Kojima H, Tsuchiya N, Tokunaga K, Masawa N, Azuma M (2004). BAFF/BLyS can potentiate B-cell selection with the B-cell coreceptor complex. Blood.

[46] Kiefer K, Oropallo MA, Cancro MP, Marshak-Rothstein A (2012). Role of type I interferons in the activation of autoreactive B cells. Immunol Cell Biol.

[47] Degauque N, Elong Ngono A, Akl A, Lepetit M, Crochette R, Giral M, Lepourry J, Pallier A, Castagnet S, Dugast E (2013). Characterization of antigen-specific B cells using nominal antigen-coated flow-beads. PLoS One.

[48] Woda M, Mathew A (2015). Fluorescently labeled dengue viruses as probes to identify antigen-specific memory B cells by multiparametric flow cytometry. J Immunol Methods.

[49] Hoh RA, Joshi SA, Liu Y, Wang C, Roskin KM, Lee JY, Pham T, Looney TJ, Jackson KJ, Dixit VP (2016). Single B-cell deconvolution of peanut-specific antibody responses in allergic patients. J Allergy Clin Immunol.

[50] Hamilton JA, Li J, Wu Q, Yang P, Luo B, Li H, Bradley JE, Taylor JJ, Randall TD, Mountz JD (2015). General Approach for Tetramer-Based Identification of Autoantigen-Reactive B Cells: Characterization of La- and snRNP-Reactive B Cells in Autoimmune BXD2 Mice. J Immunol.

[51] Titcombe P, Barsness LO, Giacobbe L, Gillespie EB, Peterson EJ, Mueller DL (2012). Production of Citrullinated Filaggrin-Specific IgG in Rheumatoid Arthritis Patients Is Associated with an Expansion of Citrullinated Filaggrin Tetramer-Binding Switched Memory Blood B Cells. Arthritis Rheum.

[52] Titcombe P, Barsness L, Giacobbe L, Gillespie E, Peterson E, Mueller D. Detection and characterization of autoreactive B cells in patients with Rheumatoid Arthritis. J Immunol. 2013;190:42.13.

